# Management of Traumatic Brain Injury: From Present to Future

**DOI:** 10.3390/antiox9040297

**Published:** 2020-04-02

**Authors:** Rosalia Crupi, Marika Cordaro, Salvatore Cuzzocrea, Daniela Impellizzeri

**Affiliations:** 1 Department of Veterinary Science, University of Messina, 98168 Messina, Italy; rcrupi@unime.it; 2 Department of Biomedical and Dental Sciences and Morphofunctional Imaging, University of Messina, Via Consolare Valeria 1, 98100 Messina, Italy; cordarom@unime.it; 3 Department of Chemical, Biological, Pharmacological and Environmental Sciences, Messina University, Viale F. Stagno D’Alcontres 31, 98166 Messina, Italy; dimpellizzeri@unime.it; 4 Department of Pharmacological and Physiological Science, Saint Louis University, Saint Louis, MO 63104, USA

**Keywords:** neuroinflammation, traumatic brain injury, palmitoylethanolamide (PEA), therapeutic strategies, oxidative stress.

## Abstract

TBI (traumatic brain injury) is a major cause of death among youth in industrialized societies. Brain damage following traumatic injury is a result of direct and indirect mechanisms; indirect or secondary injury involves the initiation of an acute inflammatory response, including the breakdown of the blood–brain barrier (BBB), brain edema, infiltration of peripheral blood cells, and activation of resident immunocompetent cells, as well as the release of numerous immune mediators such as interleukins and chemotactic factors. TBI can cause changes in molecular signaling and cellular functions and structures, in addition to tissue damage, such as hemorrhage, diffuse axonal damages, and contusions. TBI typically disturbs brain functions such as executive actions, cognitive grade, attention, memory data processing, and language abilities. Animal models have been developed to reproduce the different features of human TBI, better understand its pathophysiology, and discover potential new treatments. For many years, the first approach to manage TBI has been treatment of the injured tissue with interventions designed to reduce the complex secondary-injury cascade. Several studies in the literature have stressed the importance of more closely examining injuries, including endothelial, microglia, astroglia, oligodendroglia, and precursor cells. Significant effort has been invested in developing neuroprotective agents. The aim of this work is to review TBI pathophysiology and existing and potential new therapeutic strategies in the management of inflammatory events and behavioral deficits associated with TBI.

## 1. Introduction

Traumatic brain injury (TBI) is defined as damage to the brain sustained after the application of external physical force that causes temporary or permanent functional or structural damage to the brain. TBI stands as a major cause of death among youth in industrialized societies [1]. Brain injury can be mild, moderate, and severe. It is not a distinct unit but a heterogeneous group of pathologies that are initiated by diverse mechanisms and have different survival consequences. Head injuries can be typically classified as closed or penetrating. A closed head injury is normally used to describe automobile accidents, assaults, and falls, while a penetrating injury usually results from gunshot or stab wounds. The use of explosive devices in military conflict has generated a category known as blast injury, which is rare in injury pattern and consideration [2]. The early injury resulting from an external force creates brain tissue destruction with parenchymal impairment, intracerebral hemorrhage, and axonal cutting. Likewise, the primary insult provokes secondary neurometabolic and neurochemical events, including inflammation, cerebral edema, disruption of the blood–brain barrier (BBB), oxidative stress, excitotoxicity, and mitochondrial and metabolic dysfunctions, that can extremely modify the outcome and the recovery patterns, persisting for months to years post-injury [3]. While animal models do not replicate all the physiological, anatomical, and neurobehavioral qualities of human TBI, they do provide important insight into pathophysiological mechanisms and provide the opportunity for translational research and development of efficacious neurotherapeutic interventions [3]. Animal TBI models can be catalogued as penetrating or non-penetrating with the principal difference being the occurrence of a direct deformation of the brain in penetrating injuries, thus provoking a focal or diffused damage at the injury site. Several experimental TBI models that have been designed are listed in Table 1 [4].

The golden age of TBI research has been encouraged, thanks to the prominence of repetitive concussions or mild TBIs (mTBIs). Because of the failure of translational therapies focused on moderate to severe TBI, novel therapies have developed, defining two typical approaches. The traditional neuroprotection-based approach is based on the identification of key actions implicated in the advancement of secondary injury whether in mild or severe TBI. In this method, treatment is started as soon as possible after injury. Another methodology, more studied in clinical trials of mTBI patients, is one of targeting symptoms such as vestibular/oculomotor disturbances, headache, sleep illnesses, post-traumatic stress disorder (PTSD), cognitive dysfunction, or others [3,5]. Based on these findings, in this review, we describe the current therapeutic strategies and new therapeutic approaches for the treatment of neuroinflammatory phenomena and TBI symptom management. 

## 2. The Pathophysiology of TBI

The pathological manifestations of TBI are characterized by BBB alteration arising from cerebral ischemia, inflammation, and redox imbalances [6]. The early phase of trauma is characterized by disruption of the BBB, reduced or altered blood flow, and neuronal and glial damage [6]. Secondary injury starts from this primary injury and emerges hours, days, or months later, involves various events such as oxidative stress, modified calcium homeostasis, inflammation, and axonal damage, terminating in cellular degeneration, disturbed neural circuits, and impaired synaptic transmission and synaptic plasticity [6]. Behaviorally, these alterations manifest as post traumatic headache, depression, individuality changes, anxiety, aggression, and deficits in attentiveness, cognition, sensory processing, and communication [7,8,9].

## 3. TBI and Neurotoxicity

The neuroinflammation process that characterizes TBI progression can mobilize astrocytes, cytokines, chemokines, and immune cells toward the inflamed area, generating a pro-inflammatory reaction against insult in the brain. Nevertheless, in the chronic step, excessive activation of inflammatory mediators contributes to an injury in the brain circuit, which mainly co-occurs with secondary cell death in TBI. Different secondary cell death mechanisms drive TBI. Among these, excitotoxicity is a process characterized by increased levels of neurotransmitters and glutamate in the synaptic space that stimulate the surrounding nerve cells’ *N*-methyl-d-aspartate (NMDA) and α-amino-3-hydroxy-5-methyl-4-isoxazolepropionic acid (AMPA) receptors [10]. These receptors remain activated, favoring the influx of both sodium and calcium ions into cells [10]. In the cytosol, a high concentration of calcium ions determines the activation of protein phosphatases, phospholipases, and proteases. This activation damages DNA, structures, and membranes. In addition to apoptosis and necrosis, other forms of cell death may be active such as necroptosis, autophagy, etc. Overexcitement of glutamate receptors stimulates the production of nitrogen oxide (NO), free radicals, and pro-death transcription factors [11]. 

## 4. TBI and Oxidative Stress

Another cell death episode that happens shortly after a TBI is oxidative stress, accompanied by accumulation of both reactive nitrogen species and reactive oxygen species (RNS and ROS) [12]. High ROS levels cause lipoperoxidation of the cellular membrane, leading to dysfunction of mitochondria and oxidizing proteins, which may cause the alteration in the structure of membrane pores [13]. After the primary injury, endogenous inflammatory responses are activated with the invasion of monocytes, neutrophils, and lymphocytes through the BBB [14]. These produce prostaglandins, proinflammatory cytokines, free radicals, and several inflammatory elements, which up-regulate the levels of cell adhesion molecules and chemokines [14]. TBI activates microglia cells, which release proinflammatory cytokines and astrocytes that can up-regulate brain-derived neurotrophic factors. These, in turn, support and guide axons in recovery, increase cell production, and stimulate the long-term persistence of neurons by stopping cell death [15]. Moreover, extracellular glutamate levels are regulated by astrocytes, which also reduce glutamate excitotoxicity to neurons and other cells [16]. The pathophysiological heterogeneity detected in TBI patients may result from the nature, severity, and location of the primary injury, as well as conditions such as age, gender, genetics, and medications [17].

## 5. Biomarkers in TBI

The development of biomarkers that reveal the pathogenicity of TBI could be clinically useful to establish both diagnosis and prognosis. In particular, blood levels of the neuronal marker ubiquitin C-terminal hydrolaseL1 (UCHL1) and the astroglial marker glial fibrillary acidic protein (GFAP) represent important TBI biomarkers to support drug development and efficacy. Neurofilaments (NFs) are a major element of the axonal cytoskeleton, and play a fundamental role in structural support and regulating axon diameter [18]. Several works suggested that a phosphorylated axonal form of the heavy neurofilament (pNF-H) subunit is released from damaged neurons and might be a sensitive marker of axonal injury following TBI. In that regard, serum pNF-H was reported as a diagnostic tool to predict injury severity in patients who have suffered mild TBI, and it was helpful in understanding which patients required further CT imaging. In a recent report, simvastatin monotherapy supported neurological and functional recovery after experimental TBI, maybe via decreasing axonal injury as verified by a significant increase in the density of NF-H-positive profiles [18]. Recently, another type of pharmacodynamic/response biomarker was identified, specifically, cerebrospinal fluid (CSF) pharmaco/metabolomics are used to evaluate the influence of the combination of antioxidant N-acetylcysteine (NAC) and probenecid on the glutathione pathway after severe TBI in children [5]. Although NAC crosses the BBB, its CSF levels were only a small portion of those in blood. This is partly because NAC is speedily transported back into blood by the organic acid transporters 1 and 3. Probenecid is able to inhibit those transporters, improving brain NAC levels. Thus, the combination of probenecid and NAC produced a significant change in the CSF metabolomic markers of TBI [5]. However, the most important mTBI biomarkers are summarized in Table 2 [19]. 

## 6. Review of Existing Drug Interventions

The main contributor to secondary injury is the neuroinflammatory process principally characterized by chronic microglial stimulation, astrocytes activation, pro-inflammatory cytokines release, and oxidative stress. It was reported that it is fundamental to start the therapeutic interventions immediately following TBI, in particular within 4 h post-injury, to realize the best promising neuroprotective outcome [45]. Different therapeutic drugs with anti-inflammatory action in some experimental TBI studies are summarized in Table 3. 

In particular, minocycline, a tetracycline derivative, is pharmaceutically efficient in many models of central nervous system (CNS) illnesses and reduces inflammatory and apoptotic processes [70]. A single dose of minocycline decreases lesion volume and ameliorates neurological outcomes linked to diminished microgliosis and interleukin-1β expression in a murine model of closed head injury [59]. Administration of minocycline reduces cerebral edema and improves long-term neurological retrieval [58]. Synthetic peroxisome proliferator-activated receptor (PPAR) agonists also serve as a potent anti-inflammatory, therapeutic agents for TBI [71,72]. Fenofibrate, a PPARα receptor agonist, diminishes cerebral edema, oxidative stress, and inflammation by reducing behavioral deficits after TBI induction [60]. Pioglitazone and rosiglitazone, also PPARγ receptor agonists, diminish microglial activation, enhance neuroprotective antioxidant proteins, and change histological and behavioral outcomes after TBI [61]. Another TBI treatment approach is to block glial proliferation by cell cycle inhibition. Throughout cyclic-dependent kinase (CDK) inhibition, flavopiridol is effective at reducing lesion volume and promoting sensorimotor cognition and recovery after TBI [73]. Roscovitine, another cell cycle inhibitor, also modulates CDKs and has been shown to moderate post-injury neuroinflammation and neurodegeneration [74]. In addition, among anti-oxidants, NAC could also act as an anti-inflammatory drug. Interestingly, NAC repressed NF-κB, IL-1β, TNFα, IL-6, edema and breakdown of the BBB three days after TBI [62]. 

### 6.1. Clinical Trials of Drugs with Anti-Inflammatory Effect

Of the therapeutic strategies reported above in Table 3 for TBI management, some have already progressed into clinical trials. Erythropoietin (EPO) demonstrated potential neuroprotective proprieties in most animal models of TBI [75]. However, in a clinical trial with 200 patients presenting severe TBI, EPO administration failed to improve outcomes at 6 months [76]. Thus, although EPO has proven neuroprotective effects in preclinical animal models of TBI, its helpfulness as a medical approach is questionable [75]. In addition, a phase I/II clinical trial also showed the safety and usefulness of minocycline administration for human TBI (NCT01058395) [77,78]. Furthermore, statins, which inhibit cholesterol biosynthesis, also can promote functional recovery following TBI in rodents [79]. Simvastatin inhibits caspase-3 activation and apoptotic cell death, thereby increasing neuronal rescue after TBI [80]. Simvastatin enhances the expression of several growth factors and stimulates neurogenesis, controlling restoration of mental function [81] and supporting functional improvement after TBI (3 months) [82] in rats. However, the United States Food and Drug Administration reported cognitive side effects associated with statins treatment [83]. Given these conflicting findings, more clinical trials are needed to confirm the neuroprotective benefits of statin treatment after TBI. The effects of rosuvastatin on TBI-stimulated cytokine alteration were evaluated in a phase I/II trial (NCT00990028) [77]. 

A previous study also reported that the TNF-α antagonist, etanercept, has been given perispinally for back pain and sciatica treatment [84]. Twelve patients with chronic neurological dysfunction after TBI who were treated with etanercept showed improvements in many parameters of motor, cognitive, sensory, and psychological performance at several time points [85]. A case report also showed that a single dose of perispinal etanercept produced an important improvement in a patient with neurological dysfunction present for more than 3 years after acute brain injury [86]. Importantly, NAC also has shown potential in preventing sequelae from blast-induced mild TBI, apparently via its antioxidant capacity in the brain [87]. The safety and potential therapeutic efficacy of NAC was effectively evaluated in 41 military personnel who had a mild blast-induced TBI [87]. A phase I randomized clinical trial reported the effects of NAC in combination with an adjuvant probenecid for treatment of severe TBI in children [88]. 

Progesterone has also demonstrated helpful actions in animal models of TBI and clinical improvement in two phase II randomized, controlled trials [89]. Despite positive effects from preclinical studies and from two positive phase II clinical trials, two big phase III clinical trials of progesterone treatment of acute TBI ended with negative data, respectively, SYNAPSE (NCT01143064) and ProTECT III (NCT00822900) [89]; therefore, the results continue to fail in the field of TBI clinical trials.

### 6.2. Therapeutic Strategies to manage Neuronal Recovery and Neurobehavioral Sequelae after Injury

TBI progression affects the quality of life of a lot of people causing anxiety, agitation, memory deficiencies, and behavioral changes. Pharmacological compounds that increase cyclic 3’,5’-adenosine monophosphate (cAMP) signaling such as phosphodiesterase (PDE) inhibitors (rolipram, dipyridamole, BC11-38) [90,91], selective serotonin reuptake inhibitors (e.g., fluoxetine) [92], and serotonin-dopamine reuptake inhibitors (e.g., UWA-121), could help in brain repair, recovery of neuronal function [93], and alleviation of disabilities after injury including cognitive deficits, changes in personality, and increased rates of psychiatric illness. Table 4 gives an overview of the most frequently used treatments in the management of neuropsychiatric, neurocognitive, and neurobehavioral sequelae of injury to the brain [94].

## 7. New Therapeutic Strategies

Studying strategies to treat TBI-induced neuroinflammation requires understanding the usual mechanisms, including the tendency to protect against inflammation. Chronic inflammatory events can initiative a program of resolution that involves the release of lipid mediators capable of extinguishing inflammation [95]. Among these are fatty acid amides *N*-acylethanolamines (NAEs), including *N*-arachidonoylethanolamine (endocannabinoid), and the congeners *N*-stearoylethanolamine, *N*-oleoylethanolamine, and plus *N*-palmitoylethanolamine (PEA or palmitoylethanolamide) [96]. Several studies documented the positive effects of exogenously dispensed PEA in experimental models of TBI, spinal cord injuries, pain, cerebral ischemia, and Parkinson’s and Alzheimer diseases [97]. PEA has no unfavorable effects at pharmacological doses [97]. In addition, several experimental works showed the beneficial effects of new PEA formulations (micronized or ultramicronized) in carrageenan-induced inflammation [98] on cognitive decline associated to neuropathic pain [99] in an Alzheimer disease model [100], tibia fracture mouse model [101], and diabetic neuropathy [102]. Recent observational clinical studies reported the beneficial use of ultramicronized PEA as an add-on therapy in patients suffering from low back pain [103] as well as in patients suffering from fibromyalgia syndrome (FMS) [104]. In addition, interestingly, a co-ultramicronized PEA/luteolin (PEALUT) composite (10:1 mass ratio) displayed important neuroprotective effects in preclinical studies for various conditions (e.g., TBI, arthritis, depression, neurogenesis, Parkinson’s and Alzheimer’s diseases, and spinal cord injury) and, more recently, in experimental models of autism and vascular dementia [105,106,107,108,109,110,111,112,113]. In addition, Caltagirone et al. [114] showed that co-ultra PEALUT reduced brain injury in a rat model of Middle Cerebral Artery Occlusion (MCAO) and, more interestingly, in a clinical study. A group of 250 patients with stroke was administered a pharmaceutical formulation of co-ultraPEALut (Glialia^®^) for 60 days. At baseline and after 30 days of treatment, the patients showed improved neurological status, cognitive functions, spasticity, pain, and ability to perform activities of daily living. Despite its observational nature, the authors of [114] first described administration of co-ultraPEALut to human stroke patients, resulting in important clinical improvements. Inhibition of PEA degradation by affecting its primary catabolic enzyme, NAE-hydrolyzing acid amidase (NAAA), can also present an unconventional method to manage neuroinflammation. A recent study reported that pharmacological modulation and not blocking specific amidases for nacylamides, such as NAAA, can serve as a new approach to preserve the PEA function of maintaining cellular homeostasis through its quick, on-demand synthesis and correspondingly fast degradation. The most recent investigations reported that pharmacological changes in NAAA can be found with the oxazoline of PEA (PEA-OXA) [115]. In rat paws and the carrageenan (CAR) model, PEA-OXA accomplishes significantly better anti-inflammatory action than PEA [116]. In addition, Impellizzeri et al. [117] demonstrated the neuroprotective effects of PEA-OXA in spinal and brain injuries. PEA and new formulations of PEA, therefore, can present new therapeutic strategies in the management of neuroinflammation related to TBI and other CNS disorders.

## 8. Biologics

In addition to pharmacologic interventions for TBI, promising, innovative developments based on preclinical findings draw on the practice of biologics (e.g., gene therapy, eRNA, DNA, microRNA, antagomirs, peptide therapy, stem cells, exogenous growth factors, and peptides) [118]. Neural and mesenchymal stem cell therapy displays neuroregenerative and neurorestorative potential [119]. A recent work discussed novel associations in drug therapies that have been examined together with stem cells to overcome the restrictions allied with stem cell transplantation and to progress functional recuperation and brain repair post-TBI. To date, progesterone (clinical trials phase I and II), statins, and erythropoietin demonstrated the most encouraging results for the endogenous stem-cells-mediated repair [3]. 

Growth factors, moreover, draw significant attention for their neuroprotective and neuroregenerative efficiency. In particular, vascular endothelial growth factor (VEGF), human fibroblast growth factor 2 (FGF2), and brain-derived neurotrophic factor have been shown to improve neuronal survival when accompanying transplanted stem cells in unhealthy and injured models [120]. VEGF and FGF2 also improve functional outcomes in the preclinical model of TBI [121], while nerve growth factor decreases brain edema and reduces beta-amyloid production after TBI [122,123]. In addition, gene therapy and viral and non-viral-mediated delivery systems mark progress in managing neuronal injury. Adeno-associated viral vectors present attractive instruments for re-expressing and over-expressing genes in neurodegenerative disorders [124]. Micelles, polyethyleneimine (PEI)-coated micelles, and further micelle-like nanoparticles also might contain genetic material (DNA or RNA) and be an appealing approach for gene therapy due to their low or no immunogenicity. They can also be inserted into the brain via intranasal delivery, eliminating the need for direct intracerebral drug delivery. Nanoparticles, such as micelles, have been studied in a preclinical model of TBI to distribute DNA intranasally [125,126]. 

## 9. Neuropsychological Rehabilitation (NR) and Neurotherapy

**Figure 1:**
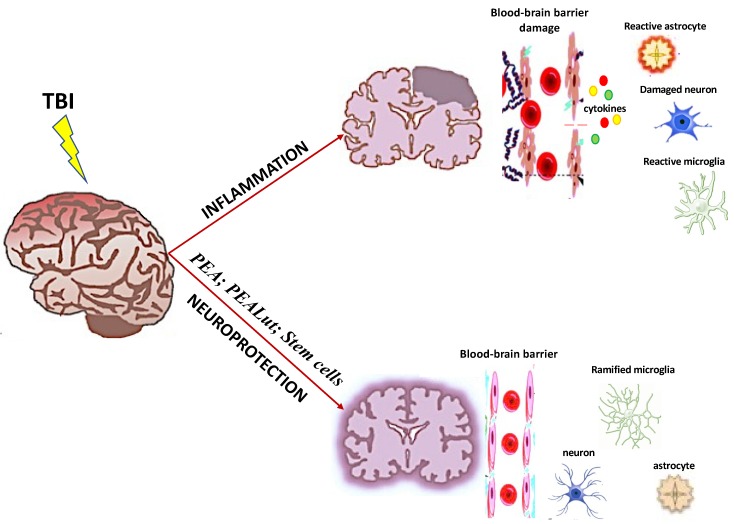
Pathophysiological heterogeneity detected in TBI.

TBI typically disturbs brain functions such as executive actions, cognitive grade, attention, memory, data processing, and language skills. Neuropsychological rehabilitation (NR) is aimed at ameliorating cognitive, emotional, psychosocial, and behavioral deficits caused by an insult to the brain. The NR of TBI patients represents a global problem, one with which modern medicine is grappling [127]. One of the central motives is the deficiency of strictly delineated theoretic supports for therapy and the means for the incessant repressing of their effects. Every brain damage causes conflicts with the so-called electric and chemical brain language, altering the space of prevailing networks and the action of neurotransmitters, which provoke a decline of the brain systems. Some studies confirmed that neurotherapy, also called neurofeedback therapy (NFT), can promote neuroplasticity [128]. NFT was shown to excite meaningful variants in structural and functional connectivity among young patients moderate TBI [127]. Transcranial magnetic stimulation (TMS) as a way of non-invasive direct modulation of neuronal activity is verysuitable for the treatment of TBI [127]. Recently, new tools for the evaluation of brain neuromarkers in TBI were developed. These include quantitative electroencephalography (EEG) to detect cortical self-regulation of the brain and event-related potentials for the flow of information in the brain [127]. Nevertheless, despite neurotherapy being very important for TBI management, more research projects are needed (Figure 1).

## 10. Conclusions

Neuroprotective approaches are the focus for TBI management, particularly methods to classify and target specific mechanisms involved in the complex secondary-injury cascade. The literature shows that neuroprotective approaches historically have been dominated by a neurocentric view, making alteration of neuronal-based injury mechanisms the primary or exclusive focus of neuroprotective strategies. The data in the literature, therefore, stress the relevance of more broadly viewing injury as comprising endothelial, microglia, astroglia, oligodendroglia, and precursor cells. Recent neuroprotection methods describe this multifaceted structure and interplay, highlighting therapeutic approaches that stimulate the recovery and optimal functioning of non-neuronal cells and inhibit the underlying mechanism of neuronal cell death. Several encouraging, recently developed treatments include neuroprotective, neurorestorative, and anti-inflammatory agents (for example PEA formulations or biologics). In addition, researchers have reported the need for developing new neurothechnologies and the neuromarkers of brain injuries to enable a correct diagnosis and, as a result, appropriate selection of methods for neuropsychological rehabilitation including neurotherapy. However, due to the difficulty and heterogeneity of brain injuries, post-TBI neural therapies are still facing several challenges.

## Figures and Tables

**Table 1:** Animal models of traumatic brain injury (TBI).

Model	Injury
FPI	Focal/diffuse
Lateral	Mixed
Middle	Mixed
CCI	Primarily focal
PBBI	Primarily focal
Blast	Primarily diffuse
Weight Drop	Focal/diffuse
Repeated Mild	Primarily diffuse

FPI: fluid percussion injury; CCI: controlled cortical impact; PBBI: penetrating ballistic-like brain injury.

**Table 2:** Biomarkers in TBI.

Biomarkers	Injury Field	Models	References
CSF/serum albumin ratio	BBB dysfunction	patients with severe TBI	[20]
Tight junction proteins		mTBI in rats ischemic stroke in rats	[21,22]
S100B		patients with minor head injury patients with extracranial pathology	[23,24]
Plasma-soluble prion protein PrPc		rat model of concussion concussed athletes	[25,26]
Tau proteins	Axonal injury	patients with acute TBI	[27]
UCHL1		patients with mild or moderate TBI patients with severe TBI concussed athletes	[28,29,30]
Neurofilaments (NFs)		rat models of TBI patients with mTBI patients with amyotrophic lateral sclerosis	[18,31,32]
NSE		Patients with severe TBI	[33]
GFAP		Rat TBI models Patients with severe TBI	[18,34]
MBP		Children with TBI	[35]
αII and βII-Spectrin breakdown products		Patients with severe TBI	[36]
NGAL		Rat model of TBI Patients with severe TBI	[37,38]
IL-6, IL-8, IL-10	Neuroinflammation	Animal and clinical models of TBI	[39,40]
MMP		mTBI patients	[41]
MBG			[42]
APOE	Genetic variations	mTBI patients	[43]
BDNF			[44]

CSF, cerebrospinal fluid; UCHL1, deubiquitinase ubiquitin carboxyl-terminal hydrolase isoenzyme L1; NSE, glycolytic enzyme neuron-specific enolase; MBP, myelin basic protein; NGAL, neutrophil gelatinase-associated lipocalin; MBG, marinobufagenin; APOE, apolipoprotein E; BDNF, brain-derived neurotrophic factor, MMP, metalloproteinase, mTBI, mild traumatic brain injury.

**Table 3:** Therapeutic drugs with anti-inflammatory action for TBI.

Drug	Route of Administration	Preclinical Model	Inflammatory Events	References
Dexamethasone	I.P.	WD	⇓ Microglia (CD68, MHC II) ⇓ Microglia (Endothelial-Monocyte Activating Polypeptide II, P2X4 Receptor, Iba-1)	[46,47]
Meloxicam	I.P.	M-WD	⇓ Lipid Peroxidation GSSH Nakatpase	[48]
Etazolate	I.P.	WD	⇓ IL-1β ⇓ Microglia (CD11b)	[49]
Carpofen	S.C.	WD	⇓ Microglia (Iba-1) ⇓ IL-1β, ⇓ IL-6 ⇔ IL-4, ⇔ IL-10	[50]
Indomethacin	I.P.	M-WD	⇓ IL-1β, ⇓ 6-Keto PGF1α	[51,52]
	I.P.	WD		
Nimesulide	I.P.	WD	⇓ 6-Keto PGF1a	[53]
Celecoxib	I.P.	WD	⇓ Il-1β, ⇔ IL-10	[52]
Meloxicam	I.P	WD	⇓ 6-Keto PGF1a	[52]
Etanercept	I.P.	FPI	⇓ TNF-α	[54]
Dexamethasone Melatonin	I.P.	CCI	⇓ MMP-2, ⇓ MMP-9, ⇓ Inos	[55]
Lipoxin A4	I.C.V.	WD	⇓ IL-1β, ⇓ IL-6, ⇓ TNFα, ⇓ GFAP ⇓ Microglia (CD11b)	[56]
Ibuprofen	I.P.	FPI	⇔ IL-4, ⇔ IL-10 ⇔ TNFα ⇔ IL-1α⇔ IL-6	[57]
Minocycline	I.P.	WD	⇓ microglia (CD11b)	[58]
	I.P.	WD	⇓ microglia, ⇓ IL-1β	[59]
Fenofibrate	P.O.	LFP	⇓ cerebral oedema ⇓ ICAM-1, ⇓ brain lesion	[60]
Pioglitazone and Rosiglitazone	I.P.	CCI	⇓ activated microglia (OX-42)	[61]
*N*-acetylcysteine	I.P.	WD	⇓ NF-kB, ⇓ IL-1β ⇓ IL-6, ⇓ TNF-α	[62]
Flavopiridol	I.P.	LFP	⇓ GFAP, ⇓ microglia	[63]
Roscovitine	I.C.V.	CCI	⇓ microglia (Iba-1)	[63]
Erythropoietin	I.P.	CCI	⇓ NF-kB, ⇓ ICAM-1, ⇓ IL-1β ⇓ TNF-α, ⇔ IL-6	[64]
	I.P.	WD	⇓ CCL-2 ⇓ microglia (CD-68)	[65]
Simvastatin	P.O.	CCI	⇓ TLR4, ⇓ NF-κB ⇓ IL-1β, ⇓ TNFα ⇓ Il-6, ⇓ ICAM-1	[66]
			⇓ Il-1β, ⇓ GFAP ⇓ IL-6, ⇓ TNF-α microglia (CD68)	[67]
Progesterone	I.P.	WD	COX-2, ⇓ PGE2, ⇓ NF-κB	[68]
	I.P./S.C.	CCI	⇓ IL-6, ⇓ COX-2, ⇓ NF-κB	[69]

⇑, increase; ⇓, decrease; ⇔, no change, I.P., intraperitoneal; S.C., subcutaneous; I.C.V., intracerebroventricular; P.O., oral; FPI, fluid percussion injury; CCI, controlled cortical impact; WD, weight drop; M-WD, Marmarou weight drop; MHC, major histocompatibility complex; CD68, cluster of differentiation protein 68; IL, interleukin; TNF, tumor necrosis factor; LFP, lateral fluid percussion; ICAM-1, intercellular adhesion molecule, MMP, metalloproteinase, COX-2, cyclooxygenase-2; NF-kB, nuclear factor-kB; GSSH, oxidized glutathione; CCL2, C–C motif chemokine ligand 2.

**Table 4:** Current drugs for neurobehavioral disorders after TBI.

Class	Drug	Mechanism	Effect
CNS stimulants	Methylphenidate	acts as an NDRI	amplified the speed of information processing in many neuropsychological tests
CNS stimulants	Modafinil	unknown	raised alertness by the modulation of both noradrenergic and dopaminergic systems
Atypical antidepressant	Agomelatine	a melatonin receptor agonist and serotonin 5-HT2C and 5-HT2B	led to better sleep efficacy
Antiviral	Amantadine	reflect a growth in synthesis and discharge of dopamine	decreased the incidence and gravity of irritability
Antidepressant of the selective SNRI class	Venlafaxine	acts as an SNDRI	increased obsessive behaviors, irritability, and sadness symptoms
Anticonvulsant	Valproate	blockade of voltage-gated sodium channels and increased brain levels of GABA	had benign neuropsychological effects, and is a safe drug to control recognized seizures or stabilize mood
Acetylcholinesterase inhibitor	Rivastigmine	inhibits butyrylcholinesterase and acetylcholinesterase	encouraging in the subgroup of patients with moderate/severe memory weakening
Cholinesterase inhibitor	Galantamine	allosteric potentiating ligand of human nicotinic acetylcholine receptors (nAChRs) α4β2, α3β4, and α6β4 and chicken/mouse nAChRs α7/5-HT3 in some brain areas	refined fatigue, memory, attention, and initiative
Cholinesterase inhibitor	Donepezil	develops cholinergic function	indorsed clinical improvement and metabolism

CNS, central nervous system; NDRI, norepinephrine–dopamine reuptake inhibitor; SNDRI, serotonin-norepinephrine-dopamine reuptake inhibitor; GABA, gamma-aminobutyric acid; SNRI., serotonin-norepinephrine reuptake inhibitor.
